# Supplementary figures and images for: The association between male involvement in institutional delivery and women’s use of institutional delivery in Debre Tabor town, North West Ethiopia: Community based survey
Source: PLoS One. 2021 Apr 9;16(4):e0249917. doi: 10.1371/journal.pone.0249917 (PMC8034730; doi:10.1371/journal.pone.0249917)

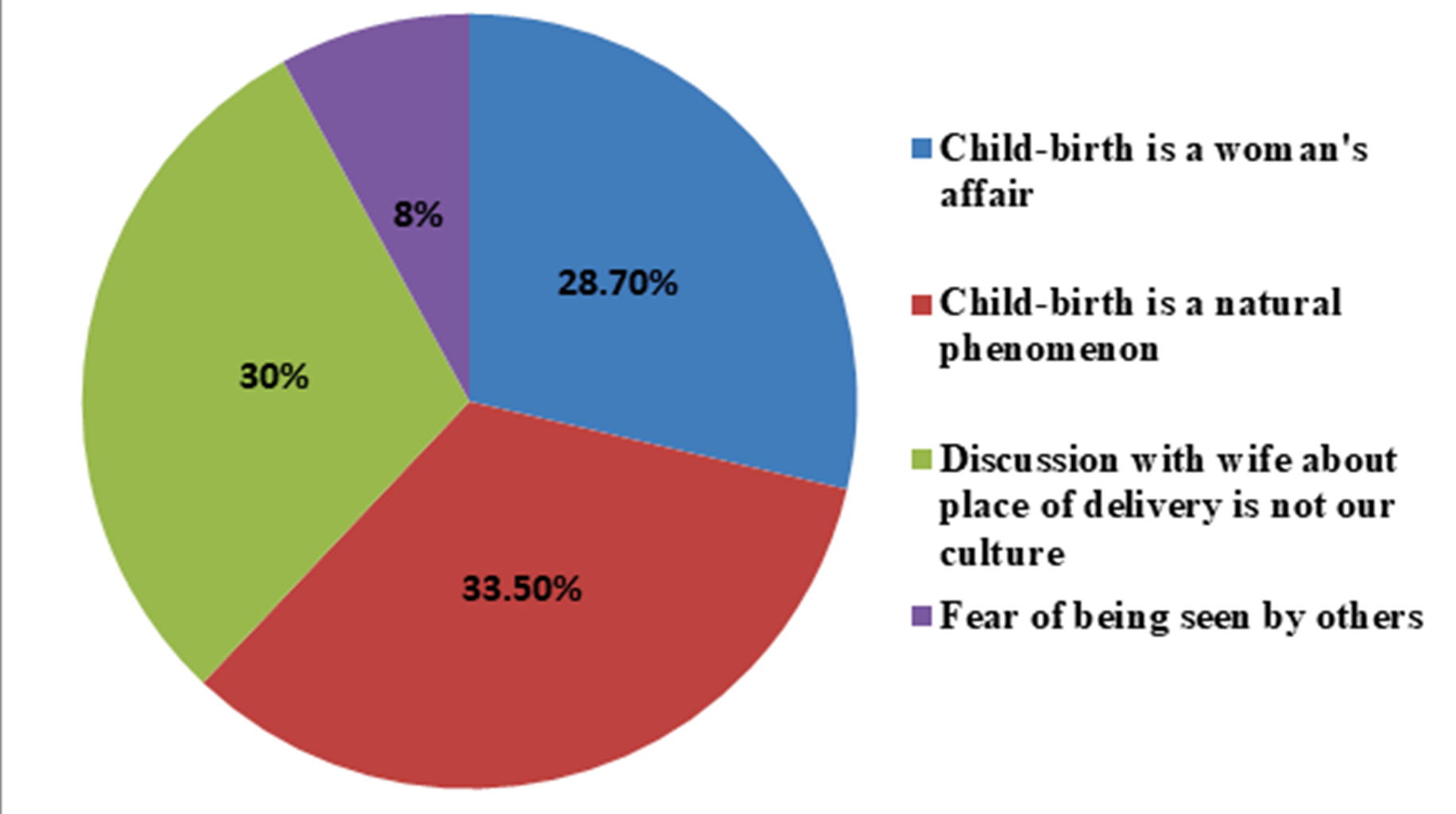

Supplement: S1 Fig — (TIF) [file pone.0249917.s001.tif]
