# Supplementary material for: The association between male involvement in institutional delivery and women’s use of institutional delivery in Debre Tabor town, North West Ethiopia: Community based survey
Source: PLoS One. 2021 Apr 9;16(4):e0249917. doi: 10.1371/journal.pone.0249917 (PMC8034730; doi:10.1371/journal.pone.0249917)
Supplement: S1 Table — (PDF) [file pone.0249917.s002.pdf]

S1 Table: Male partners knowledge on maternal health in Debre Tabor town, North-West Ethiopia, 2019 (n=477)

Variables	Frequency	Percent
**Do you know about your spouse ANC follow up**		
Yes	290	60.9
No	187	39.1
**Minimum number of ANC a women should attend**		
One	8	2.3
Two	60	17.0
Three	155	44.0
Four and more	254	53.2
**Institutional delivery is important to get skilled attendants**		
Yes	115	24.1
No	362	75.9
**Institutional delivery prevents delay in getting medical care in a case of emergency**		
Yes	70	14.7
No	407	85.3
**Institutional delivery is important to get immediate treatment for mother and new born**		
Yes	124	25.9
No	353	74
**Do you know why institutional delivery is important**		
Yes	331	69.4
No	146	30.6
**Vaginal bleeding is a sign of complication during pregnancy**		
Yes	276	57.9
No	201	42.1
**Fever is a sign of complication during pregnancy**		
Yes	134	28.1
No	343	71.9
**Abdominal pain is a sign of complication during pregnancy**		
Yes	114	23.9
No	363	76.1
**Difficulty in labor is a sign of complication during pregnancy**		
Yes	48	10.1
No	429	89.9
**Convulsion is a sign of complication during pregnancy**		
Yes	101	21.2
No	376	78.8
**Do you know the signs of pregnancy complication**		
Yes	417	87.4
No	60	12.6
**The above complication during labor and child birth could lead to death**		
Yes	284	59.5
No	193	59.5
**Overall Knowledge Score**		
Good	165	34.6
Poor	312	65.4
