# Supplementary material for: The association between male involvement in institutional delivery and women’s use of institutional delivery in Debre Tabor town, North West Ethiopia: Community based survey
Source: PLoS One. 2021 Apr 9;16(4):e0249917. doi: 10.1371/journal.pone.0249917 (PMC8034730; doi:10.1371/journal.pone.0249917)
Supplement: S2 Table — (PDF) [file pone.0249917.s003.pdf]

S2 Table: Male partners attitude towards institutional delivery in Debre Tabor town, North-West Ethiopia, 2019 (n=477)

Variables	Response	Frequency	%
**Believe that pregnant women are susceptible to face delivery complication**	Agree	175	36.6
	Disagree	302	63.4
**Delivery complication can be hazardous for pregnant women**	Agree	189	39.6
	Disagree	288	60.3
**Delivery complication can be hazardous for new born**	Agree	137	28.7
	Disagree	340	71.3
**Do you believe being attended by a skilled delivery attendant may be beneficial for spouse**	Agree	198	41.5
	Disagree	279	8.5
**Believe that being attended by skilled attendant beneficial for newborn wellbeing**	Agree	148	31
	Disagree	329	69
**Safe place for delivery is the needs of pregnant woman**	Agree	209	43.8
	Disagree	268	56.2
**Attitude score**	Favourable	234	49.1
	Unfavourable	243	50.9
