# Supplementary material for: The association between male involvement in institutional delivery and women’s use of institutional delivery in Debre Tabor town, North West Ethiopia: Community based survey
Source: PLoS One. 2021 Apr 9;16(4):e0249917. doi: 10.1371/journal.pone.0249917 (PMC8034730; doi:10.1371/journal.pone.0249917)
Supplement: S1 File — (PDF) [file pone.0249917.s004.pdf]

# Annex I: Information Sheet

**Title of the Research Project**: male involvement in institutional delivery and its associated factors among married male who have children less than one year of age in Debre Tabor town, North West, Ethiopia 2019.

**Name of Principal Investigator**: Kassanesh melese

**Name of the Organization**: Bahirdar University College of Medicine and Health Science Department of Reproductive health.

**Name of the Sponsor**: Self sponsor

Information Sheet is prepared for participants who have included in the study during data collection.

**Introduction**

This information sheet is prepared with the aim of assessing male involvement in institutional delivery and its associated factors among married male who have children less than one year of age in Debre Tabor town, North West, Ethiopia 2019.

**Purpose of the Research Project**: The aim of this study is to assess male involvement in institutional delivery and its associated factors among married male in Debre Tabor town, North West, Ethiopia 2019. The results of this study was used to design appropriate intervention programs to address the problems related to male involvement in institutional delivery in Amhara Region, Northwest Ethiopia.

**Procedure**: The study involves men who have children less than one year of age had fulfilled the criteria of male involvement in institutional delivery and its associated factors among married male in Debre Tabor town, North West, Ethiopia 2019. You are selected to be one of the study participants if you are willing to take part in this study and we kindly invite you to take part in our project. If you are willing to participate, we are so happy and we need you to clearly understand the aim of this study and show your agreement. Finally, you are kindly requested to give your genuine response in the interview.

**Benefits, Risk and /or Discomfort**

By participating in this research project, you may feel some discomfort in wasting your time (a maximum of 30 minutes).

However, your participation is definitely important to identify the factors associated with male in institutional delivery among married male in Debretabor Town North West Ethiopia 2019. There is no risk or direct benefit in participating in this research project.

**Incentives/Payments for Participating**

You will not be provided any incentives or payment to take part in this project.

**Confidentiality**

The information collected from you will be kept confidential and stored in a file, without your name by assigning a code number to it. Hence, no report of the study ever identifies you.

**Right to Refusal or Withdraw**

You have the full right to refuse from participating in this research. You have also the full right to withdraw from this study at any time you wish.

**Person to contact**

This research project will be reviewed and approved by the ethical committee of Bahirdar University College of medicine and health sciences. If you have any question you can ask any time.

Name of contact person: Kassanesh melese

Tele: (+251)918715018 or (251)963711703

E-mail: mkassu5018@gmail.com

# Annex II: Consent Form

## Introduction

Good morning/afternoon, my name is ______________ I am working with Kassanesh Melese who is doing a research for the partial fulfillment of Master's Degree at Bahir Dar University. The questioner is intended to asses' male involvement in institutional delivery and its associated factors among married malein Debre Tabor town, North West, Ethiopia 2019.

Your truth full answers for all of our questions about factors associated with home delivery among women will be very important. Your answers will be confidential and secret. If you decide that, you do not want to participate in the study, or that decide at any time in the future you do not want to participate. I appreciate if you try to answer all the questions. If you agree to participate, we will go 30 minutes for us to complete the questionnaire. If you have any questions about the study, you can ask.

Thank you. Next, I will read a consent, which assures your interest to participate.

The researcher explained the aim of the study. Moreover, to decide any time if I do not want to participate. Therefore, I assure that my interest to participate in this study is truly from my knowledge.

If client refuses, please check this box ☐

Signature of Person administering consent_______________

Date______________________

Client's Signature_____________

Date_____________________

Identification number: ___________________________

Data collector (Name & Number): ______________________

## Annex III. English version questionnaires for interview

### Identification Information

001. Code No.________ 002. Kebele____ Date of interview________

**Section 1:** socio-demographic characteristics of male partners

| No | Question | Option | |
|---|---|---|---|
| 01 | Age in year | ________ | |
| 02 | Religion | 1.Orthodox<br>2. Muslim<br>3. Protestant<br>4. Catholic<br>5. Other specify | |
| 03 | Ethnicity | 1. Oromo<br>2. Amhara<br>3. Tigre<br>4. Guragie<br>5. Others | |
| 04 | Occupation | 1. Civil servant<br>2. Private employee<br>3. Farmer<br>4. Merchant<br>5. Other | |
| 05 | Educational status | 1.Illiterate | |

| | | | |
|---|---|---|---|
| | | 2 .Able to read and write<br>3. Primary education<br>4.Secondary education<br>5. Certificate and diploma<br>6.Degree and above | |
| 06 | Monthly income of the family | ___________________ | |

**Section 2:** Questions aboutmale involvement in institutional delivery**(Health service related factors, cultural believe and practices)**

| | | | |
|---|---|---|---|
| 01 | Have you ever gone to health facility with your spouse for ANC checkup in her previous<br>pregnancies? | 1.Yes<br>2.No | If No skip Question number 02 |
| 02 | If so, how many times did you go? | 1.One<br>2.Two<br>3.Three<br>4.Four<br>5.Above four | |
| 03 | Did you prepare for delivery when?<br>your wife got pregnant with your | 1.Yes<br>2.No | If No skip Question number 04 |

| | | | |
|---|---|---|---|
| | youngest child? | | |
| 04 | If so, what preparations did you make for the delivery | 1.Saved money for delivery<br>2.Arranged for transport<br>3.Planned ahead for a place of delivery<br>4.Identified a person who follow her in the health facility for delivery<br>5.Prepare essential items for delivery<br>6.Diffirent food item preparation<br>7.Other (Specify)____________ | |
| 05 | Have you had discussions with Health care provider regarding the place of delivery during your spouse pregnancy | 1.Yes<br>2.No | If No skip question number 06 |
| 06 | What was your motive to discuss? with health Professionals about pregnancy related issue and place of delivery? | 1.My previous experiences<br>2.When she feels pain<br>3.my interest<br>4.Others (specify)___________ | |
| 07 | Have you ever had discussion with | 1 yes | If No skip |

| | | | |
|---|---|---|---|
| | anyone concerning the place of delivery of your wife | 2. No | question number 08 |
| 08 | If yes, with whom did you have the discussion with (more than one answer are permissible) | 1.Relative<br>2.Friends<br>3. Other (specifies)____________ | |
| 09 | what was their suggestion while?<br>your spouse was pregnant for<br>Place of delivery? | 1.They encouraged me to take her health institution<br>2.They discourage me to take<br>health institution<br>3.They gave no suggestions<br>4.I don't remember | |
| 10 | Where did your spouse give birth?<br>for your youngest child | 1.Health facility<br>2.At home | |
| 11 | Decision making regarding about the place of delivery? | 1.male partners with spouse jointly<br>2.Your spouse<br>3.male partners<br>4.Others(specify) _____________ | |
| 12 | Is there accessibility of delivery? | 1.Yes | If No skip |

| | | | |
|---|---|---|---|
| | site. (yes, if less than 30 minutes walking, no if more than 30 minutes walking) | 2.No<br>3.I don’t know | question number 13 |
| 13 | If yes which site is more than accessibly for you if need arise? | 1.Hospital<br>2.Health center<br>3.Private clinics | |
| 14 | What was your opinion about the cost of accessing health facility Delivery? | 1.Absolutely free<br>2.Partially free<br>3.Affordable<br>4.Expesive<br>5.Very expensive | |
| 15 | If ans. to Q 14 is No.4 and 5 In Which Health facility to reach? | 1.Governmental hospital<br>2.Health center<br>3.Private clinic | |
| 16 | Have you ever gone to health facility with your spouse for delivery? | 1.yes<br>2.No | If No skip question number 17 |
| 17 | If yes what was you dislike about your nearest health facility from | 1.Distance covered to access skilled care | |

| | | | |
|---|---|---|---|
| | the past delivery history. (more than one answer is possible) | 2.Attitude/behavior of health staff<br>3.Opening hours (day/night)<br>4.Service provider readiness (bed, water, medicine)<br>5.Cost<br>6. Other specify_____________ | |
| 18 | What do you think about the attitude of health workers towards male who accompany their wives to health facility to seek care? | 1.Cooperative and welcoming<br>2. Uncooperative and harsh<br>3.Do not know | |
| 19 | What traditional beliefs do you think makes male partners not to involve themselves in the decision of their delivery place (more than one answer is possible) | 1.Child-birth is a woman's affairs that does not require men participation<br>2.Child-birth is natural phenomenon that should not be given much attention<br>3.It's not our culture to discuss with wife about place of delivery<br>4.Fear of being seen by others<br>5.Other (specify)___________ | |

**Section 3:** Questions about knowledge of male involvement towards institutional delivery

| | | | |
|---|---|---|---|
| 20 | Do you know your spouse have Received ANC follow up? | 1.Yes<br>2.No | If No skip to Q 24 |

| | | | |
|---|---|---|---|
| | | | |
| 21 | What is the recommended minimum number of times that a pregnant woman needs to attend ANC | 1.0nce<br>2.Twice<br>3. Three times<br>4.Four times | |
| 22 | What do you think about the importance of institutional delivery (more than one answer possible) | 1.Access to skilled care<br>2.prevent delay in getting emergency care if needed<br>3.Immediate treatment to the mother and baby<br>4.I don’t know | |
| 23 | In your opinion, what are some serious health problems that can occur during labor and childbirth that could endanger the life of a pregnant woman? (more than one answer possible) | 1.Vaginal bleeding<br>2.Fever<br>3.Abdominal pain<br>4.Diffcult in labor<br>5.convulsion<br>6.I don’t know<br>**7.**Other(Specify)_______________ | |
| 24 | In your opinion, could a woman die from (this problem) any of these | 1.Yes<br>2.No | |

| | | | |
|---|---|---|---|
| | Problemsmentioned above? | | |
| 25 | Do you know what you help your wife during delivery? | 1. Yes<br>2. No | |
| 26 | If YES to Q No 26, what were the ways you helped her during delivery? | 1. Took her to deliver<br>2. Encouraged her through the process<br>3. Made sure she was handled by a trained health worker<br>4. Did all she asked me to.<br>5.Others(specify)__________________ | |
| 27 | If NO to Q No 26 why? | 1. Not male's role<br>2. Not allowed in our local culture<br>3.Not aware of what a male needed to do<br>4.Not allowed by women to participate<br>5.Too busy with work<br>6. Feel ashamed/embarrassed<br>7. Don't know/not sure<br>8.Others(specify)________________ | |
| 28 | What do you think are some of the reasons why male have not been involved the way they ought to have been? | 1.Lack of sufficient knowledge on how to be more involved<br>2.Restrictions due to culture and tradition<br>3.Restrictions from health | |

| | | | |
|---|---|---|---|
| | | facilities/workers<br>4.Restrictions by women themselves<br>5. No program to encourage male to be more involved<br>6. Insufficient money to do what we may want to do<br>7.Others (specify)___________ | |

**Section 4:** questions about male partner attitude towards institutional delivery.

| | | | |
|---|---|---|---|
| 29 | Do you believe Any pregnant women are susceptible to face delivery complication? | 1.Strongly disagree<br>2.Disagree<br>3. Neutral<br>4. Agree<br>5.Strongly agree | |
| 30 | Delivery complication can be sever and may be hazardous for pregnant woman. | 1.Strongly disagree<br>2.Dis agree<br>3. Neutral<br>4. Agree<br>5.Strongly agree | |
| 31 | Delivery complication can be sever and hazardous for newborn | 1.Strongly disagree<br>2.Dis agree<br>3. Neutral<br>4. Agree | |

| | | | |
|---|---|---|---|
| | | 5.Strongly agree | |
| 32 | Do you believe being attended by a skilled delivery attendant may be<br>Beneficial for spouse. | 1.Strongly disagree<br>2.Disagree<br>3. Neutral<br>4. Agree<br>5.Strongly agree | |
| 33 | Do you believe being attended by a skilled delivery attendant may be<br>Beneficial for newborn wellbeing. | 1.Strongly disagree<br>2.Disagree<br>3. Neutral<br>4. Agree<br>5.Strongly agree | |
| 34 | Do you believesafe place for delivery is the needs of a pregnant woman immediately before and during the time of delivery? | 1.Strongly disagree<br>2.Disagree<br>3. Neutral<br>4. Agree<br>5.Strongly agree | |

**Annex IV፡**አማርኛመጠየቅየመጀመሪያየስምምነትውልበባህርዳር ዩኒቨርሲቲ ጤና ሳይንስ ት/ቤት የስነ-ተዋልዶ ጤና ትምህርት ክፍል

*አማርኛ መጠየቅ የመጀመሪያ የስምምነት ውል ቅፅ*

***ሀ. መግቢያ***

ጤና ይስጥልኝ! ________________ እባላለሁ፡፡ የምሰራው በባህርዳር ዩኒቨርሲቲ የስነ ተዋልዶ ጤና ሳይንስ ትምህርት ክፍል የሁለተኛ ዲግሪ ማሟያ ጥናታዊ ፅሑፍ/ምርምር/በማድረግ ላይ ከሚገኙት ካሳነሽ መለሰ ከተባሉ ጥናት አድራጊ ጋር ስሆን፣በደብረታቦር ከተማ ውስጥ ያሉ ወንድ የትዳር አጋሮች በሰለጠነ ባለሙያ በሚሰጥ የወሊድ አገልግሎት ያላቸዉተሳትፎ በተመለከተ ለማወቅ ቃለ መጠይቅ እያደረግሁኝ እገኛለሁ፡፡ እርስዎም የጥናቱ ተሳታፊ ይሆኑ ዘንድ ተመርጠዋል፡፡

**የምርምሩ/ጥናቱርዕስ**

በደብረታቦር ከተማ ያሉ ወንድ የትዳር አጋሮች በሰለጠነ ባለሙያ በሚሰጥ የወሊድ አገልግሎት እንዳይሳተፉ የሚያደርጉ ምክንያቶችን ለማወቅ የሚካሄድ ጥናት ነው፡፡

**የጥናቱዓላማ**፡-ከአንድ አመት በታች የሆኑ ህፃናት ያላቸዉ ወንድ የትዳር አጋሮች በሰለጠነ ባለሙያ በሚሰጥ የወሊድ አገልግሎት ላይ ያላቸዉ ተሳትፎ በተመለከተ ለማወቅና ከዚህ ጋር ተያያዥነት ያላቸውን ዋናዋና ጉዳዮችን በመሰብሰብ በሰለጠነ ባለሙያ በሚሰጥ የወሊድ አገአገልግሎቱን ና ፕሮግራሙን ይበልጥ ለማሻሻል ሲሆን ፡ሌላው በህብረተሰብ ጤናሳይንስ የሁለተኛ ዲግሪ ማሙያ ፅሑፍ ለማቅረብ ነው፡፡

**አተገባበር**፡-ከላይ የተመለከተውን ጥናት ለማካሄድ የተለያዩ ጥያቄዎች ይኖሩናል ፡፡ጥናቱ ውጤታማ ሊሆን የሚችለው እርሶ በሚሰጡት ትክክለኛ መልስ ላይ በመሆኑ ጥያቄዎቹን በጥንቃቄ እንዲመልሱልን ፍቃደኝነትዎን በትህትና እንጠይቃለን፡፡ ግልፅ ያልሆነልዎትን /እንዲብራራልዎት/ የሚፈልጉት ጉዳይ ካለ መጠየቅ ይችላል፡፡

**የጥናቱ ጥቅምና ጉዳት**፡-እርሶ በዚህ ጥናት ተሳታፊ በመሆንዎ በቀጥታ ሊያገኙ የሚችሉት ነገር ላይኖር ይችላል፣ ነገርግን የእርሶ ተሳትፎ በጥናቱ አላማ ወሊድ በሰለጠነ ባለሙያ የወንዶች ተሳትፎ ዙሪያ ያለውን ክፍተት ለማሳየት እናት ክክለኛ የመፍትሔ አቅጣጫ ለመጠቆም እጅግ በጣም አስፈሊጊ ነው፡፡ በዚህ ጥናት በመሳተፎ ምንም አይነት ጉዳት አይደርስቦትም፡፡ በመጠይቁ ውስጥ ለመመለስ የማይፈልጉት ጉዳይ ካለም ምላሽ እንዲሰጡ አይገደዱም፡፡

**ምስጢራዊነት**፡-ለዚህ ጥናት/ ፕሮጀክት/ የሚሰበሰብ ማንኛውንም ዓይነት መረጃ ምስጢራዊነቱ የተጠበቀ ሲሆን የርስዎም ስም ሳይፃፍበት ስውር ሚስጥራዊ ቁጥር ብቻ ተሰጥቶት በፋይል ውስጥ የሚቀመጥ ይሆናል እንዲሁም መረጃው ጥናቱን ከሚያካሂደው ሰው በስተቀር ለማንም ዓይነት ሰው ግልፅ አይሆንም፡፡

**ተሳትፎ**፡-በዚህ ጥናት ላይ መሳተፍ /አለመሳተፍ/ ሙሉ በሙሉ በርስዎ ፈቃደኝነት ላይ የተመሰረተ ነው፡፡ ለጥያቄዎቹ በሙሉም ሆነ በከፊል መልስ ያለመስጠት መብት አለዎት፡፡ ይህ

ደግሞ ማንኛውንም አይነት ግልጋሎት ከማግኘት አያግድዎትም፡፡ እንዲሁም በፈለጉት ሰዓት ማንኛውንም መብትዎን ሳያጡ የማቋረጥ ሙሉ መብት አለዎት፡፡

የበለጠ መረጃ ካስፈለጎት የሚከተለትን አድራሻ መጠቀም ይችላሉ

ጥናቱን የሚያካሂደው ሰው አድራሻ፡ ካሳነሽ መለሰ ባህርዳር ዩኒቨርሲቲ ስነተዋልዶ ጤና ትምህርት ክፍል

እርሰዎ በዚህ ጥናት ላይ ለመሳተፍ ፈቃደኛነዎት?

አዎን፡- ወደ ቃለ መጠይቁ ይሂዱ፡፡

አይደለሁም፤አመሰግናለሁ፡፡ወደ ሚቀጥለው ተሳታፊ ይሂዱ፡፡

የቃለ መጠይቅ አቅራቢው ስም ______________________________ ፊርማ ____________

ቃለ መጠይቅ የተደረገበት ቀን __________________

**Annex Vየአማርኛመጠይቅ**

ቀበሌ_________የተጠያቂው የኮድ ቁ_____የመጠይቅ ቀን__________

**ክፍልአንድ፦-ማህበራዊና ኢኮኖሚያዊ ሁኔታ የሚዳስሱ ጥያቄዎች**

| ኮድ | ጥያቄዎች | ለጥያቄው መልስ ሊሆን የሚችለው | ወደ ቀጣይ እለፍ |
|---|---|---|---|
| 01 | ዕድሜ? | _________ | |
| 02 | ሃይማኖት? | 1.ፕሮቴስታንት<br>2.ኦርቶዶክስ<br>3.ሙስሊም<br>4.ካቶሊክ<br>5.ሌላካለ (ይጥቀሱ)_________ | |
| 03 | ብሔር? | 1.አማራ<br>2.ትግሬ<br>3.ኦሮሞ<br>4.ጉራጌ<br>5.ሌላ ካለ(ይጥቀሱ) | |
| 04 | ሥራ? | 1.የመንግስት ድርጅት ተቀጣሪ<br>2.የግልድርጅት ተቀጣሪ<br>3.ነጋዴ<br>4.ገበሬ<br>5.ሌላ ካለ(ይጥቀሱ)________ | |
| 05 | የትምህርት ደረጃ | 1.ያልተማረ<br>2.ማንበብና መጻፍ የሚችል<br>3.አንደኛ ደረጃ ያጠናቀቀ<br>4.ሁለተኛ ደረጃ ያጠናቀቀ | |

| | | | |
|---|---|---|---|
| | | 5. ሰርተፍኬት እና ዲፕሎማ<br><br>6.ዲግሪ ና ከዚያ በላይ | |
| 06 | የቤተሰብወርሃዊ ገቢ | _________ | |

**ክፍል 2: በጤናተቋምውስጥለሚሰጥየወሊድአገልግሎትየወንድ/የትዳርጓደኛተሳትፎበተመለከተ ለጥናቱተሳታፊዎችየተዘጋጀመጠይቅ**(Health service related factors and cultural believe and practices)

| | | |
|---|---|---|
| 01 | ከትዳርጓደኛዎ ጋርለቅድመወሊድክትትልወደጤናተቋምሄደዋልበቀድሞውእርግዝናወቅት? | 1. አወ<br>2. አልሄድኩም |
| 02 | አዎከሆነምንያህልጊዜያትሄዱ? | 1. አንድ<br>2. ሁለት<br>3. ሶስት<br>4. አራት<br>5. ከአራትበላ[illegible] |
| 03 | ሚስትህታናሹንልጅ<br>ባረገዘችበትወቅትለወሊድተዘጋጅተህነበር | 1. አዎ<br>2. አልተዘጋ[illegible] |
| 04 | አዎ ከሆነምንዝግጅቶችተዘጋጅተዋል? | 1. ለወሊድገ[illegible]<br>2. ለትራንስ[illegible]<br>3. የወሊድአ[illegible]<br>4. በጤናተቋ[illegible]<br>5. ለወሊድአ[illegible]<br>6. የተለያዩየ[illegible]<br>7. ሌላ_____ [illegible] |

| | | |
|---|---|---|
| 05 | በባለቤትወእርግዝናወቅት የወሊድ ቦታዉን<br>በተመለከተእርስዎከጤናባለሙያጋርተወያይተዉነበር | 1. አዎ<br>2. አልተወያ[illegible] |
| 06 | ከጤናባለሙያዎችጋርለመወያየትያነሳሳህነገርምንነበር? | 1. የቀድሞል[illegible]<br>2. ህመም [illegible]<br>3. በራሴፍላ[illegible]<br>4. ሌሎች ([illegible] |
| 07 | የባለቤትውንየወሊድቦታበተመለከተእርስዎተወያይተዉያዉቃሉ | 1. አወ<br>2. አልተወያ[illegible] |
| 08 | አዎከሆነ ውይይትያደረጉትከእነማንጋርነው | 1. ዘመድ<br>2. ጓደኞች<br>3. ሌሎችዝር[illegible]<br>(ከአንድበላይመል[illegible] |
| 09 | የትዳርጓደኛዎእርጉዝእያሉ<br>ስለወሊድቦታዉበተመለከተየእነርሱሀሳብምንነበር? | 1. ወደጤናተ[illegible]<br>2. ወደጤናተ[illegible]<br>3. እነሱምንግ[illegible]<br>4. አላስታው[illegible] |
| 10 | የትዳርጓደኛህ<br>ትንሽልጅዎንየትወለደች | 1. ጤናተቋም[illegible]<br>2. ቤትውስጥ[illegible] |
| 11 | የወሊድቦትውንበተመለከተዉሳኔያችሁ እንዴት ነበር | 1. የትዳርአጋ[illegible]<br>2. የትዳርጓደ[illegible]<br>3. አንተነህ<br>4. ሌሎች |
| 12 | የጤና ተቋም ተደራሽነት አለ (አዎ ከሆነ 30 ደቂቃዎች ያነሰ የሚራመዱ,የለም ከሆነ 30 ደቂቃዎች በላይ በእግር የሚያስጉዝ) | 1. አዎ<br>2. የለም<br>3. እኔ አላው[illegible] |
| 13 | አዎ ከሆነ የትኛው ተቋም ነው ለእርስወ የሚቀርበዉ | 1. የመንግስት[illegible]<br>2. ጤና ጣቢ[illegible]<br>3. የግል ክሊ[illegible] |

| | | |
|---|---|---|
| | | |
| 14 | የጤናአገልግሎትተደራሽነትወጪ፣በእርስዎአመለካከትምንይመስላል? | 1. በፍፁምነ[illegible]<br>2. በከፊልነፃ [illegible]<br>3. ተመጣጣ[illegible]<br>4. ዉድ<br>5. በጣምውድ[illegible] |
| 15 | ለጥያቄቁጥር14 ቁጥር 4 እና 5 ንከመረጡየትኛውየጤናተቋም ለመድረስ? | 1. የመንግስ[illegible]<br>2. ጤናጣቢ[illegible]<br>3. የግልክሊ[illegible] |
| 16 | ከትዳርጓደኛዎ ጋርለወሊድ አገልግሎት ወደጤናተቋምሄደዋል? | 1. አወ<br>2. አልሄድኩ[illegible] |
| 17 | መልስወ አወ ከሆነ በአቅራቢያዎ የሚገኘውን ጤና ተቋም የማይወዱት ለምንድን ነዉ የትዳር ጓደኛዎ የመጨረሻ ልጅዎን ሲወልዱ ያጋጠመዎት ችግር ላይ የተመሠረተ መልስ ይስጡ (ከአንድ በላይ መልስ ሊኖር ይችላል) | 1. ባለሙያ[illegible]<br>2. የጤና ባለ[illegible] አሰጣጥ<br>3. የስራ ሰዓ[illegible]<br>4. የጤና አገ[illegible] ባለመሙ[illegible]<br>5. የክፍያ ሁ[illegible]<br>6. ሌላ ካለ [illegible] |
| 18 | ወንዶች ሚስቶቻው የጤና እክብካቤ አገልግሎት ለማግኘት አብረው ወደ ጤና ተቋም ቢሄዱ የጤና ባለሙያዎች በወንዶቹ ላይ ያላቸዉ እይታ/ባህሪያት ምንድን ነው ብለው ያስባሉ? | 1. የሚተባበ[illegible]<br>2. የማይተባ[illegible]<br>3. አላውቅም |
| 19 | ምንዓይነትባህላዊእምነቶች \የወንዶችበወሊድቦታላይእንዳይሳተፉ ያደርጋቸዎልብለውያስባሉ (ከአንድበላይመልስሊገኝይችላል) | 1. ልጅመው[illegible]<br>2. ልጅ- መውለድ[illegible] ይገባ በመ[illegible]<br>3. የወሊድቦ[illegible] |

| | | |
|---|---|---|
| | | በተመለከተ[illegible]<br>4. ሌሎችእን[illegible] |

ክፍል 3: የትዳር አጋሮች በጤና ተቋም የወሊድ አገልግሎት ላይ ያላቸዉን እዉቀት በተመለከተ የሚዳስሱ ጥያቄወች (KNOWLEDGE)

| | | | |
|---|---|---|---|
| 20 | የትዳር ጓደኛዎ የቅድመ ወሊድ ክትትል ማግኘቷን ታውቃለህ | 1. አዎ<br>2. አላዉቅም | አላዉቅም ከሆነ ጥያቄ ቁጥር 22 ን ይለፉ |
| 21 | አንዲት ነፍሰጡር ሴት የቅድመ ወሊድ ክትትል ቢያንስ ምን ያህል ጊዜ ያስፈልጋታል | 1. አንድ<br>2. ሁለት<br>3. ሶስት<br>4. አራት | |
| 22 | የወሊድ አገልግሎት በጤና ተቋም ለምን አስፈላጊ ይሆናል ብለህ አሰብክ (ከአንድ በላይ መልስ ሊኖር ይችላል) | 1. የአገልግሎት ተደራሽነት ሠለጠነ ባለሙያ ስለሚሰጥ<br>2. አስፈላጊ ከሆነ የድንገተኛ ጊዜ ጤና እንክብካቤ መዘግየትን ለመከላከል<br>3. ለእናት እና ለህፃኑ አፋጣኝ መፍትሄ ለመስጠት<br>4. እኔ አላውቅም | |
| 23 | በአንተ ግንዛቢ በምጥ እና ወሊድ ጊዜ የሚከሰቱ ህይወትን ሊያሳልፉ የሚችሉ ከባድ የጤና ችግሮች የሚባሉት ምን ምን ናቸዉ (ከአንድ በላይ መልስ ሊኖር ይችላል) | 1. ደም መፍሰስ<br>2. ትኩሳት<br>3. የሆድ ህመም<br>4. የምጥ መዘግየት/ከባድ ምጥ<br>5. መንቀጥቀጥ<br>6. እኔ አላውቅም | |
| 24 | በነዚህ ከባድ የጤና ችግሮች ምክኒያት እናት ህይወቷ ሊያልፍ ይችላል ብለህ ታስባለህ | 1. አዎ<br>2. አይደለም | |

| | | | |
|---|---|---|---|
| 25 | ባለቤትህ ስትወልድ ምን መርዳት እንዳለብህ ታዉቃለህ | 1. አዎ<br>2. አላዉቅም | |
| 26 | ለጥያቄ ቁጥር 26 መልስዎ አወ ከሆነ በምን መልኩ ነዉ ያገዝካት | 1. የወሊድ አገልግሎት ወደ ሚሰጥበት ተቋም በመዉሰድ<br>2. በሂደቱ ውስጥ አገዝኩት<br>3. በሰለጠነ ባለሙያ እንደምትረዳ አረጋገጥኩ<br>4. የምትጠይቀኝን ሁሉ በማድረግ<br>5. ሌሎች ካሉ ይጥቀሱ_________ | |
| 27 | ለጥያቄ ቁጥር 26 መልስዎ አላዉቅም ከሆነ ለምን | 1. የወንዶች ሀላፊነት ስላልሆነ<br>2. ባህላችን ስላልሆነ<br>3. ወንድ ማድረግ ያለበትን ግንዛቢ ስላልነበረኝ<br>4. ወንዶች እንዲሳተፉ ሴቶች ስለማይፈቅዱ<br>5. ስራ በጣም ስለሚበዛብኝ<br>6. ሀፍረት ስለተሰማኝ<br>7. ምክኒያቱ ምን እንደሆነ አላቀዉም | |
| 28 | ለጥያቄቁጥር 33 መልስዎአይደለምከሆነእንዳይሳተፉያደረጋቸዉምክኒያትምንድነዉብለህታስባለህ | 1. ተሳትፎ ለማድረግ በቂ የሆነ የእዉቀት እጥረት ስላለ<br>2. የባህል እና የልማዶች ገደብ መኖር<br>3. በጤና ተቋማት/ባለሙያዎች ክልከላ ስላለ<br>4. በ ሴቶች ክልከላ ስላለ<br>5. ወንዶች በበቂ ሁኔታ እንዲሳተፉ የሚያደርግ አሰራር ባለመኖሩ<br>6. ማድረግ የምችለዉን ለማድረግ በቂ ገንዘብ ስለለኝ<br>7. ሌሎች ካሉ ይጠቀስ_________ | |

## ክፍል 4፡የወንዶች ዝንባሌ የሚመለከቱ መጠይቆች (Attitude)

| | | | |
|---|---|---|---|
| | በእርስዎ እምነት ነብሰ ጡርእናትበወሊድወቅትለ | 1. በጣምአልስማማም | |

| | | | |
|---|---|---|---|
| 29 | ከባድየጤናችግርየተጋለጠችናትብለዉያስባሉ | 2. አልስማማም<br>3. አላዉቀዉም<br>4. እስማማለሁ<br>5. በጣምእስማማለሁ | |
| 30 | ከወሊድጋርተያይዞየሚመጣከባድየጤናችግርለነብሰጡር እናት አደገኛእናአስጊይሆናል | 1. በጣምአልስማማም<br>2. አልስማማም<br>3. አላዉቀዉም<br>4. እስማማለሁ<br>5. በጣምእስማማለሁ | |
| 31 | ከወሊድ ጋር ተያይዞ የሚመጣ ከባድ የጤና ችግር ለህጻኑ አደገኛ እና አስጊ ይሆናል | 1. በጣምአልስማማም<br>2. አልስማማም<br>3. አላዉቀዉም<br>4. እስማማለሁ<br>5. በጣምእስማማለሁ | |
| 32 | በእርስዎ እምነት በሠለጠነባለሙያየሚሰጥየወሊድ አገልግሎት ለእናትጤንነት ጠቃሚይሆናል | 1. በጣም አልስማማም<br>2. አልስማማም<br>3. አላዉቀዉም<br>4. እስማማለሁ<br>5. በጣም እስማማለሁ | |
| 33 | በእርስዎ እምነት በሠለጠነ ባለሙያ የሚሰጥ የወሊድ አገልግሎት ለህጻኑ ጤንነት ጠቃሚ ይሆናል | 1. በጣም አልስማማም<br>2. አልስማማም<br>3. አላዉቀዉም<br>4. እስማማለሁ<br>5. በጣም እስማማለሁ | |
| 34 | አስተማማኝ የሆነ የወሊድ ቦታ<br>ለነብሰጡር እናቶች በወሊድ ሰአት ከወሊድ በፊትና በሁላ ያስፈልጋታል ብለህ ታምናለህ | 1. በጣምአልስማማም<br>2. አልስማማም<br>3. አላዉቀዉም<br>4. እስማማለሁ<br>5. በጣምእስማማለሁ | |

**ስለተሳትፎዎ*በጣም* አመሰግናለሁ!!!**
