# Supplementary material for: The association between male involvement in institutional delivery and women’s use of institutional delivery in Debre Tabor town, North West Ethiopia: Community based survey
Source: PLoS One. 2021 Apr 9;16(4):e0249917. doi: 10.1371/journal.pone.0249917 (PMC8034730; doi:10.1371/journal.pone.0249917)
Supplement: Attachment — Submitted filename: PONE-D-20-32675.docx [file pone.0249917.s006.docx]

Thank you for the opportunity to review this manuscript

An interesting study drawing on the views of local participants to understand why men do not regularly participant in MCH care. It's strengths are a clear presentation of findings, however it lacks clarity and detail in relation to the rationale for the study, methods used and implications of the findings. There are minor grammatical errors and typos throughout.

Introduction:

More detail needed on the possible impact of increased male partner involvement. Are there studies which show the impact that increased male involvement can have? What does male involvement look like (e.g. attending appointments, births etc)?

The necessity of the study is in question. Still feel like the background does not provide for a clear problem statement. Together, I would suggest the authors during their write up to consider using recent references especially those from countries with similar maternal mortality rates to Ethiopia.

Why is a study in Debre Tabor town important? Can the authors tell us about maternal and child morbidity/mortality rates there? How might the findings be relevant to the international audience? This is referred to later but a line or two at the end of the introduction would clarify the importance of the study and allow the reader to determine whether the paper is relevant to them.

Methods:

Please clarify how was male involvement defined?

The description of the outcome variable is not clear. It would be important to clarify that the variable was computed based on responses to [number of items] items: as listed and having at least 6 times [provide a comprehensive list]. It would also be important to clearly state that men who responded yes to n or more items were classified as being “? involved” while those who respondent yes to n-1 or fewer items were classified as being “less ? involved”. If there is a citation to support the coding of the variable in this way, it should be provided.

Results:

Needs to be re-written as it's extremely long, check language issues in the tables as well as avoid replicating results into the discussion section. Revisions In the tables, the total N should be included in each table. In all tables, are multiple responses included? Some editing is required

Discussion

Their interpretation of the implications of this finding is also flawed – the discussion seems a bit repetitive with more focus on restating the findings rather than discussing implications for the findings or possible future directions related research could take based on the findings. If the authors can be clearer about the added value of their study, or the importance for their setting. It would help to have a clear idea of the purpose behind the current objectives. The similarities and differences of this study needs to be brought out more clearly so that the reader can see how it adds to the current evidence. The discussion section should also include methodological issues or limitations.

The study does not give clear implications in the conclusion or recommendations. If the study provides similar findings as other studies, where is the problem? What is the explanation for this? What should be done about this situation? What should be the next logical step to address this issue? Recommendations for practice and future research should be included.

I noted that you used different reference styles so kindly use only PLOS ONE reference style through out the entire manuscript

Please provide a copy of the interview questionnaire used in this study as an additional file.
