# Supplementary material for: The association between male involvement in institutional delivery and women’s use of institutional delivery in Debre Tabor town, North West Ethiopia: Community based survey
Source: PLoS One. 2021 Apr 9;16(4):e0249917. doi: 10.1371/journal.pone.0249917 (PMC8034730; doi:10.1371/journal.pone.0249917)
Supplement: Attachment — Submitted filename: ethiopia-male.docx [file pone.0249917.s007.docx]

This paper seems to be a fair summary of literature on **Male partners’ involvement in promoting institutional delivery and its effect on the**

**actual place of delivery in Debre Tabor town, North West Ethiopia: community based survey** Methods of identifying relevant studies for review are thoroughly explained. My only concerns are as follows;

- An English editorial correction is needed
- No scientific concern noted in then article.
