# Supplementary material for: The association between male involvement in institutional delivery and women’s use of institutional delivery in Debre Tabor town, North West Ethiopia: Community based survey
Source: PLoS One. 2021 Apr 9;16(4):e0249917. doi: 10.1371/journal.pone.0249917 (PMC8034730; doi:10.1371/journal.pone.0249917)
Supplement: Attachment — Submitted filename: Response to Reviewers.docx [file pone.0249917.s008.docx]

**The association between male involvement in institutional delivery [and women’s use of institutional delivery in Debre Tabor town, North West Ethiopia: community based survey](https://bmcpregnancychildbirth.biomedcentral.com/articles/10.1186/s12884-019-2342-3)**

**Authors:** Kassanesh Melese Tessema, Kebadnew Mulatu Mihirete, Endalkachew Worku Mengesha, Azezu Asres Nigussie, Awoke Giletew Wondie

To: Dr. Sara Ornaghi
Academic Editor

PLOS ONE

**Subject: Revised version of the manuscript (PONE-D-20-32675)**

Dear Editor,

We would like to extend our gratitude for the academic editor and the reviewers for providing constructive comments which we believe it will further improve the quality of the manuscript. We carefully reviewed all the comments and provided a point-by-point response for them. Moreover, we marked the changes in a tracked-change version of the manuscript.

At this juncture, *the authors received no specific funding for this work.* We believe that this manuscript fits very well within the scope of PLOS ONE since it covers important aspects of maternal and child health. Hope the revisions will satisfy the academic editor and reviewers concerns.

With regards,

Awoke Giletew Wondie

Corresponding Author

**Authors’ response to Editor**

**Comment**

1. Please ensure that your manuscript meets PLOS ONE's style requirements, including those for file naming.

Response: Corrected as per PLOS ONE's style requirements.

2. We suggest you thoroughly copyedit your manuscript for language usage, spelling, and grammar. If you do not know anyone who can help you do this, you may wish to consider employing a professional scientific editing service.

Response: We consulted expert editor for language usage, grammar and typo error checking. The revised version of the manuscript is edittted thoroughly.

3. In the Methods, please discuss how the questionnaire was validated and pre-tested. If these did not occur, please provide the rationale for not doing so.

Response: We have expanded our earlier statement related to data validation and pre-teste. In the revised manuscript page 8 lines 116-124, we presented as follows:

“As a data quality assurance measure, a pre-test was conducted on about 10% of the sample that has similar characteristics with the study population other than the sampled households in the study area before actual data collection period. The appropriateness and clarity of questions were checked and necessary modifications were made in terms of clarifying questions and vague terms, and removing items that were not aligned with the study objectives. Each item in the questionnaire was checked by the supervisor and the principal investigator on a daily basis to review the accuracy and completeness of variables. At the completion of data collection, all entered records were inspected for missing or incorrect values and data cleaning and coding was conducted before analysis by the principal investigator.”

4. In your discussions and conclusions please take care to avoid statements implying causality from correlational research. For example, avoid the use of terms such as "predictors/ predictions" or “effect” or “resulted in." Instead consistently use terms such as "associated with" or "associations.

Response: Thank you. We have now amended it accordingly.

5. Please upload a new copy of Figure 1 as the detail is not clear. Please follow the link for more information:

Response: We have now uploaded Figure 1 again based on PLOS ONE's style requirements.

**Response to Reviewers**

**Reviewer #1: An interesting study, however it lacks clarity and detail in relation to the rationale for the study, methods used and implications of the findings. There are minor grammatical errors and typos throughout.**

Response: We consulted expert editor for grammar and typo error checking. The revised version of the manuscript is edittted thoroughly.

**Introduction**

1. More detail needed on the possible impact of increased male partner involvement. Are there studies which show the impact that increased male involvement can have?

Response: We have incurporated a statement on the possible impact of increased male partner involvement. In the revised manuscript page 4 lines 43-50, we presented as follows:

“High male involvement stemming from knowledge of delivery process results in positive perception towards skilled birth care and involvement manifested through such actions as physical and emotional and financial support which will lead to good maternal health outcomes [21]. These practice has implications for maternal health as it determines the nutritional status of women during pregnancy [22]; women’s access to maternal health services since healthcare systems in most developing countries require out-of-pocket payments [23]; and women’s chances of receiving emergency obstetrics care, which is vital in averting maternal mortality [22].”

Additionally, a specific benefits of male involvement is mentioned in page 5 lines 55-58.

2. What does male involvement look like (e.g. attending appointments, births etc)?

Response: Thank you. We made a clear description of what male involvement mean and the extent of male involvement in maternal healthcare in the introduction and method section of revised manuscript accrodignly. In the introduction we have incorporated the following revision and presented as follows (page 4 lines 30-38):

“However, the term ‘male involvement’ is subjective and very multifaceted [16]. Most studies defined male involvement as the active participation of men in maternal health services/care (attending maternal health service and support/help); while others defined it as providing financial support for pregnancy and childbirth related expenses. The remaining studies defined male involvement as shared decision-making on maternal health with wife [17]. In this study male involvement in institutional delivery is operationalized as the active participation of men in: accompanying for ANC, birth preparedness, encouraging for institutional delivery, and discussion with spouse, relatives and health professionals on place of delivery.”

3. The necessity of the study is in question. Still feel like the background does not provide for a clear problem statement. Together, I would suggest the authors during their write up to consider using recent references especially those from countries with similar maternal mortality rates to Ethiopia.

Response: Apologies for the confusion and we have now amend the whole introduction section again and clarified the statement of problem using more recent references.

4. Why is a study in Debre Tabor town important? Can the authors tell us about maternal and child morbidity/mortality rates there?

Response: Even though specific data on maternal and child morbidity/mortality rates in in Debre Tabor town is not available, however, correction is made by incorporating country wide data in the introduction section (page 3 lines 7-15).

5. How might the findings be relevant to the international audience? This is referred to later but a line or two at the end of the introduction would clarify the importance of the study and allow the reader to determine whether the paper is relevant to them.

Response: Thank you. For international audience, despite several studies reported the benefit of male involvement however, it’s impact on maternal health service utilization is not clear and well investigated in developing countries where the greatest burden of global maternal deaths and men’s dominant roles in these countries have been shown to influence health outcomes.

This correction is made on the last paragraph of introduction on the revised manuscript (page 5 lines 61-73) and presented as follows:

“Evidence suggests that male involvement may be beneficial to maternal health; however, the magnitude of the association is not clear and whether involving men in maternal health can improve maternal outcomes remains unclear [17, 30, 31]. There have also been speculations on possible negative impacts of male involvement in maternal health, hence it is necessary to explore the impact of male partner’s involvement on the female partner’s use of maternal health services in developing countries where the greatest burden of global maternal deaths and men’s dominant roles in these countries have been shown to influence health outcomes. In Ethiopia where maternal death, males economic dominance and decision-making power remained high, attempts to examine male partner’s involvement and its role on maternal health service utilization have been very limited, relying largely on socio-demographic and maternal characteristics [32, 33].”

**Methods**

6. Please clarify how was male involvement defined? The description of the outcome variable is not clear. It would be important to clarify that the variable was computed based on responses to [number of items] items: as listed and having at least 6 times [provide a comprehensive list]. It would also be important to clearly state that men who responded yes to n or more items were classified as being “? involved” while those who respondent yes to n-1 or fewer items were classified as being “less ? involved”. If there is a citation to support the coding of the variable in this way, it should be provided.

Response: We have now rewrite it as suggested clearly in the revised manuscript page 8 lines 126-133, presented as follows:

“Male involvement in institutional delivery was our primary outcome variable computed based on responses to six items: accompanying spouse for ANC, participating in birth preparedness plan, encouraging spouse for institutional delivery, discussion with health professionals on the place of delivery, discussion with relatives and friends about the place of delivery. Therefore, men who responded “yes” to 3 or more items were cat­egorized as “involved” while those who responded “yes” to less than 3 items were cat­egorized as “not involved”. Moreover, prior studies on male involvement in institutional delivery applied this approach of categorization [34-38].”

**Results**

7. Needs to be re-written as it's extremely long, check language issues in the tables as well as avoid replicating results into the discussion section. Revisions In the tables, the total N should be included in each table. In all tables, are multiple responses included? Some editing is required.

Response: We have now re-write the result section shortly and some phrases in the tables were modified. Additionally, we have included the total n in each table, and multiple responses were considered in Table 6 (showing the result of male partners involvement in institution delivery).

**Discussion**

8. The discussion seems a bit repetitive with more focus on restating the findings rather than discussing implications for the findings or possible future directions related research could take based on the findings. The similarities and differences of this study needs to be brought out more clearly so that the reader can see how it adds to the current evidence.

Response: We have now ameneded the discussion section again by discussing the implications of the findings for possible future directions and edittted for language usage, gramattic error thoroughly. The similarities and differences of this study main findings are mentioned clearly in the revised manuscript so that the reader can see how it adds to the current evidence. Additionally, the corrections made for these issues is summarized at the end of discussion in the revised manuscript page 19 lines 327-335, presented as follows.

“Previous studies mostly focused on the positive benefits of male involvement in maternal and child health; however, the magnitude of the association is not clear. This study assesses the impact of male partner’s involvement on the female partner’s use of institutional delivery. Our findings showed that active participation of men appeared to have more impact on women’s utilisation of institutional delivery. This study also reinforces the findings on the significant influence of education, knowledge and attitude on maternal health and socio-cultural barriers on male involvement in institutional delivery. The impact of male partner’s involvement on maternal and childbirth outcomes, how men could have better involvement and the time of involvement (during pregnancy, delivery and postpartum period) merit further investigations.”

9. The discussion section should also include methodological issues or limitations.

Resonse: Thank you. The limitation of this study is clearly indicated in the last paragraph of discussion section in the revised manuscript page 19 lines 336-342.

10. The study does not give clear implications in the conclusion or recommendations. What should be done about this situation? What should be the next logical step to address this issue? Recommendations for practice and future research should be included.

Response: This study reported a significant beneficial impact of male involvement on maternal health through improved utilisation of institutional delivery. It implies the need to shift from women-only maternal health services to ‘male-friendly’, couple services, and also to improve healthcare/government policies that isolate/discourage men from having active engagement in maternal health. We have now amended these issues and detail is provided in the conclusion section of revised manuscript page 19-20 lines 334-353.

11. I noted that you used different reference styles so kindly use only PLOS ONE reference style through out the entire manuscript.

Response: Apologies or the situation and we have now fix it.

12. Please provide a copy of the interview questionnaire used in this study as an additional file.

Response: In the revised submission, we have included the study questionnaire as a supplementary file named as S1_File.pdf.

**Reviewer #2: This paper seems to be a fair summary of literature on Male partners’ involvement in promoting institutional delivery and its effect on the actual place of delivery in Debre Tabor town, North West Ethiopia: community based survey Methods of identifying relevant studies for review are thoroughly explained. My only concerns are as follows;**
An English editorial correction is needed,

No scientific concern noted in then article.

Response: We consulted expert editor for language usage, grammar and typo error checking. The revised version of the manuscript is edittted thoroughly.

**Reviewer #3: As the authors note, many studies on maternal healthcare utilization exclude men, and I find this piece novel and refreshing for its attention to men’s perspectives and experiences. That said, I have several suggestions the authors might consider in order to strengthen the manuscript**.

**Introduction**

1. To start, it would be helpful for them (Authors) to define what is mean by the term “male partner involvement” in this context. Does it refer to men attending ANC appointments, being present at time of delivery, arranging transport and payment of clinic fees, etc., or does “involvement” go beyond this rather narrow, biomedical framing?

Response: Thank you. We made a clear description of what male involvement mean and the extent of male involvement in maternal healthcare in the introduction and method section of revised manuscript accrodignly. In the introduction we have incorporated the following revision and presented as follows (page 4 lines 30-38):

“However, the term ‘male involvement’ is subjective and very multifaceted [16]. Most studies defined male involvement as the active participation of men in maternal health services/care (attending maternal health service and support/help); while others defined it as providing financial support for pregnancy and childbirth related expenses. The remaining studies defined male involvement as shared decision-making on maternal health with wife [17]. In this study male involvement in institutional delivery is operationalized as the active participation of men in: accompanying for ANC, birth preparedness, encouraging for institutional delivery, and discussion with spouse, relatives and health professionals on place of delivery.”

2. How are men expected to ensure women’s and children’s health? Second, could you be clearer as to why and how male involvement/support is so important for female reproductive health? You mention the role of men in decision-making regarding healthcare seeking, but could you say more?

Response: We have expanded the statement on why and how male involvement/support is so important for female reproductive health. In the revised manuscript page 4-5 lines 39-50, we presented as follows:

In most developing countries, the decision to seek care is usually made by male partners [18] and they are the chief providers which often determining women’s access to economic resources [19]. Similarly in Ethiopia, women traditionally enjoy little independent decision making on most individual and family issues, including the option to choose whether to give birth in a health facility or seek the assistance of a trained provider [20]. High male involvement stemming from knowledge of delivery process results in positive perception towards skilled birth care and involvement manifested through such actions as physical and emotional and financial support which will lead to good maternal health outcomes [21]. These practice has implications for maternal health as it determines the nutritional status of women during pregnancy [22]; women’s access to maternal health services since healthcare systems in most developing countries require out-of-pocket payments [23]; and women’s chances of receiving emergency obstetrics care, which is vital in averting maternal mortality [22].

Additionally, a statement on the specific benefit of male partner involvement is included in the revised manuscript page 5 lines 55-58.

3. The article you cite stating the lack of evidence on this theme is from 2012 and I think you’ll see that since this time, quite a bit has been published on the topic of male partner involvement, including a number of intervention studies and those assessing factors that either encourage/discourage men from becoming involved in this arena.

Response: Thank you. In the original manuscript we mentioned that evidence on how maternal health care utilization is affected by participation of their husbands is lacking in both developing and developed countries. We have now re-write it again after revising recent papers. In the revised manuscript page 5 lines 61-67, we presented as follows:

“Evidence suggests that male involvement may be beneficial to maternal health; however, the magnitude of the association is not clear and whether involving men in maternal health can improve maternal outcomes remains unclear [17, 30, 31]. There have also been speculations on possible negative impacts of male involvement in maternal health, hence it is necessary to explore the impact of male partner’s involvement on the female partner’s use of maternal health services in developing countries where the greatest burden of global maternal deaths and men’s dominant roles in these countries have been shown to influence health outcomes.”

4. I would also recommend that you contextualize the issue within Ethiopia a bit more. What is the burden of maternal and child morbidity/mortality in this context? What factors influence poor reproductive health outcomes, including low rates of institutional delivery?

Response: The burden of maternal mortality, lower rates of maternal health service utilization and other factors influencing maternal health sevice use, and the gender role of men including thier economic dominance and decision-making power in Ethiopia are incorporated in different paragraphs of introduction section. Such as Page 3 lines 7-15; Page 4 lines 24-26, 41-43; Page 5 lines 52-54 and 67-71.

**Methods**

5. As for your methods section, there are some important details that are missing and/or unclear. For instance, in your abstract you mention that the study included married men who have children less than one year of age, but in the Population section of your Methods, you just indicate “all male partners.” How are you defining “male partner”? This is an important distinction to make, as I would imagine marital status would influence the extent of men’s involvement/influence on women’s reproductive health.

Response: Thank you. We also consider marital status would influence the extent of men’s involvement/influence on women’s reproductive health. Men in marital union with a women (legal, religious or traditional) was considered as male partner. It was a typo error but it has now corrected in the method section of revised manuscript page 6 lines 82-85, as follows:

“The source population was all men in marital union with a women (legal, religious or traditional) having children less than one year of age and permanent residents of Debre Tabor town. All men in marital relationship having children less than one year of age and permanent residents of Debre Tabor town and selected during the study period were our study population.”

6. Additionally, I am wondering why you chose only to include men with children one year of age and below? Does this have to do with recall bias?

Response: Yes, recall bias could present as pregnancy and child birth may not represent as much significance to the male partner as it does to the female. We mentioned this as one limitation of study in the revised manuscript page 19 lines 336-342.

7. Where were data collected – i.e. were participants interviewed in their homes or within a health facility?

Response: Data were collected through house to house visit as it is a community based survey. It is indicated in the method section of revised manuscript page 7 line 107.

8. What was the specific random sampling technique utilized (how did you choose where to start) and what kind of systematic technique was used thereafter?

Response: After allocating the sample size proortionall for each kebeles and calculating a sampling interval, the study subjects were selected by systematic random sampling using every kth interval. The first father was selected by lottery method and subsequent fathers were included randomly based on the interval through house to house visit. Detail is provided on method section of revised manuscript page 6-7 lines 94-103.

9. When you mention that data were collected using a questionnaire adopted from previous studies (pg. 6, line 79) can you cite these previous studies or say more about them? Also, how were they adapted? Did adaptation go beyond simple translation, and if so, how?

Response: It was a typo error but it has now corrected. The questionnaire was adapted from the survey tools developed by African Medical and Research Foundation (AMRF), Child and Reproductive Health Programme which is modified according to local context. Additionally, after pre-testing, the appropriateness and clarity of questions were checked and necessary modifications were made in terms of clarifying questions and vague terms, and removing items that were not aligned with the study objectives. All these corrections are incorporated in method section of revised manuscript page 7 lines 108-111 and page 8 lines 118-120.

**Discussion**

10. As currently written, it contains much repetition of your findings, and also mostly focuses on how your findings are similar to those of other studies.

Response: We have now ameneded the discussion section again by discussing the implications of the findings for possible future directions and edittted for language usage, gramattic error thoroughly.

11. I would like to see you emphasize more that which differentiates your study. What new knowledge does it add to the literature?

Response: Previous studies mostly focused on the positive benefits of male involvement in maternal and child health; however, the magnitude of the association is not clear. The new things in this study is it assesses the impact of male partner’s involvement on the female partner’s use of institutional delivery. Our findings showed that active participation of men appeared to have more impact on women’s utilization of institutional delivery. This study also reinforces the findings on the significant influence of education, knowledge and attitude on maternal health and socio-cultural barriers on male involvement in institutional delivery. The impact of male partner’s involvement on maternal and childbirth outcomes, how men could have better involvement and the time of involvement (during pregnancy, delivery and postpartum period) merit further investigations. These corrections was made on the entire discussion section, additionally these issues were summarized at the end of discussion in the revised manuscript page 19 lines 327-335.

12. And going a step further, I think you could speak more specifically to the implications of your findings for policy and programming. Clearly, you can conclude that interventions promoting greater institutional delivery would do well to involve men, but you could also speak to why and how men could be better involved.

Response: Yes, this study reported a significant beneficial impact of male involvement on maternal health through improved utilisation of institutional delivery. It implies the need to shift from women-only maternal health services to ‘male-friendly’, couple services, and also to improve healthcare/government policies that isolate/discourage men from having active engagement in maternal health. We have now amended these issues and detail is provided in the conclusion section of revised manuscript page 19-20 lines 334-353. Furthermore, how men could be better involved is recomeneded for further studies in the discussion section of revised manuscript.

13. Your findings regarding joint decision making by couples seem really important here; perhaps interventions should strive to be more gender inclusive?

Response: Yes it is. Some studies define male involvement as a shared descision making with wife on maternal and child health issues which is not similar with our operational definition. However, this study reported that majority of spouse give birth in heath institution (60%) for which majority of them made a joint descision on the place of delivery (60%). We have mentioned this in the discussion section as one possible explanation for the strong association between male involvement and women’s use of institutional delivery in page 17 lines 287-294 of revised manuscript.

14. Further, how might policies be enacted to help address some of the barriers to men’s participation that you’ve identified in this study? Do you think other barriers – beyond knowledge and attitude – might need to be addressed?

Response: Yes, the most frequently mentioned socio-cultural barriers for male involvement (considering child birth as a natural phenomenon that should not be given much attention and their culture did not allow them to discuss with wife about place of delivery) should be addressed. Therefore, maternal health interventions should target husbands as con­sumers of maternal health services and efforts should be made to improve awareness of the male partner’s on institutional delivery through community based health education. All these issues were described in the discussion and conclusion section of manuscript accordingly.

15. What about time and cost-related factors that may prevent men from becoming more involved in the biomedical encounter? Again, I think this comes back to the question of defining what “involvement” means, as I mentioned earlier.

Response: Thank you. Despite the definition used for male involvement in institutional delivery in this study (accompanying spouse for ANC, birth preparedness, encouraging for institutional delivery and discussion with spouse, relatives and health professionals on place of delivery) have no time and cost realted variables, however, these variables were included as an explanatory variable when we identify factors asscated with male involvement in institutional delivery and no significant association was obtained.

16. On a smaller note, you say that men’s involvement in maternal health is “culturally discouraged and remains an important barrier” (pg. 17, line 246) but then you cite a study from Nepal. Can you speak to what current literature from sub-Saharan Africa suggests, and particularly those studies conducted in Ethiopia? Why is men’s participation socially discouraged in this context?

Response: We have now change this statement in the revised manuscript, and literatures in Ethiopia and sub-Saharan Africa were used. Men’s participation was socially discouraged mainly due to socio-cultural barriers including a belife that pregnancy and child birth issues are solely women’s issue.

17. Around line 282 you mention men may fear “being ruled” by their wives. Where does this explanation come from, can you cite this?

Response: Similarly we have now change this statement in the revised manuscript, and the reference used for this statement is replaced by literatures in Ethiopia and sub-Saharan Africa.

18. Further, I would encourage you to consider how gender norms are not static but constantly changing. Is there any evidence to suggest that gender norms around male participation in the female reproductive sphere are shifting? And if so, in light of what factors?

Response: Yes, evidence is emerging that men and boys around the world are increasingly accepting gender equality and more equitable norms – particularly younger men, those with higher levels of education, and those in urban areas. But change toward gender equality also brings resistance. There is also a growing evidence base of interventions that have, in relatively limited spaces, shown signs of achieving change. The key to gender norm change is to provoke it and speed it up from within, acknowledging and forming alliances with those men and boys who already believe in gender equality, and identifying the conditions necessary to scale programs up in schools, the workplace, the health sector and other spaces where millions can be reached. All of this must be supported by public policies that reinforce gender equality and include penalties for those who violate rights. It must also include efforts to assess both the implementation and impact of gender equality policies, including efforts to engage men and boys in norm change.

19. Finally, there are a substantial number of issues relating to grammar or awkward wording that require editing. I would highly recommend careful editing during the revision process, perhaps in conjunction with a professional copyeditor. This is needed, as it’s disrupting the flow of what is otherwise an important study.

Response: We consulted expert editor for language usage, grammar and typo error checking. The revised version of the manuscript is edittted thoroughly.

**Minor Comments**

20. Pg. 3, line 2 (Introduction) – the first sentence does not make grammatical sense. Try “in which many women are exposed”

Response: We revised this statements, and now appeared in page 3, lines 2-3.

21. The next two sentences of the Introduction need citations.

Response: Corrected.

22. Pg. 3, line 10 – I would specify that you mean 85% of *maternal deaths

Response: Yes, we revised this statements and now appeared in page 3, lines 9.

23. Pg. 7, lines 95-97: I would recommend referring your readers to Table 4 here in case they are wondering how you’re measuring “knowledge about maternal health.” The same goes for “partner attitude” with respect to Table 5.

Response: Thank you. For short and brief presentation of the findings for readers, we have no refered these tables which could be accessed in the supplementary files.

24. Pg. 8, line 124: Could you contextualize respondents’ average monthly income for those of us unfamiliar with Ethiopian currency? Are participants able to meet their daily needs with this level of income?

Response: Yes, those who earn 1000 ETB can meet their daiy needs. Households above 1000 ETB monthly income are characterized by the number and kinds of consumer goods they own (ranging from a television to a bicycle or car), and the housing characteristics such as source of drinking water (pipe, public tap/standpipe, tube well, spring and rain water); and flooring materials (sand, dung, wood, palm/bamboo, ceramic and cement).
